# Endogenous electric fields as guiding cue for cell migration

**DOI:** 10.3389/fphys.2015.00143

**Published:** 2015-05-13

**Authors:** Richard H. W. Funk

**Affiliations:** Institute of Anatomy, Technische Universität-DresdenDresden, Germany

**Keywords:** endogenous, electric fields, cell, migration, wound healing, regeneration, embryonic development

## Abstract

This review covers two topics: (1) “membrane potential of low magnitude and related electric fields (bioelectricity)” and (2) “cell migration under the guiding cue of electric fields (EF).”Membrane potentials for this “bioelectricity” arise from the segregation of charges by special molecular machines (pumps, transporters, ion channels) situated within the plasma membrane of each cell type (including eukaryotic non-neural animal cells). The arising patterns of ion gradients direct many cell- and molecular biological processes such as embryogenesis, wound healing, regeneration. Furthermore, EF are important as guiding cues for cell migration and are often overriding chemical or topographic cues. In osteoblasts, for instance, the directional information of EF is captured by charged transporters on the cell membrane and transferred into signaling mechanisms that modulate the cytoskeleton and motor proteins. This results in a persistent directional migration along an EF guiding cue. As an outlook, we discuss questions concerning the fluctuation of EF and the frequencies and mapping of the “electric” interior of the cell. Another exciting topic for further research is the modeling of field concepts for such distant, non-chemical cellular interactions.

## Introduction

Each cell (not only neural cells!) produces a membrane potential that is specific for its type and tissue and which is also specific for its degree of differentiation. It normally varies from −40 mV to −90 mV in differentiated cells and from −8.5 mV in the fertilized egg, −23 mV in the four-cell and −25 mV in the 16-cell frog embryo (see Binggeli and Weinstein, 1986; Levin, 2007). Interestingly, tumor cell membrane potentials are also in the range of the embryonic cells (Chernet and Levin, 2013; Funk, 2013).

The electric nature of these membrane potentials producing endogenous electric fields (EF, direct currents, DC and ultra-low frequency electromagnetic fields, UL-EMF: see also Funk et al., 2009), comes from the segregation of charges by molecular machines like pumps, transporters and ion channels that are mostly situated in the plasma membrane (for reviews see Simanov et al., 2012). Ions and charged molecules can also pass from cell to cell by gap junctions and by this a gradient is produced and again an EF. It must be emphasized that these endogenous EF are steady or slowly changing gradients. They have lower membrane potential values than the typical action potentials in nerve or muscle cells (10–20 V/cm are needed for stimulation with surface electrodes), and do not show spikes of electric activities but smooth potentials that can change over a longer period (days till weeks—e.g., during wound healing) (McCaig et al., 2005) and even form UL-EMF. It is an intrinsic property of any cell to generate these potentials of low magnitude.

In epithelial layers of the early embryo, for example, currents and related fields are actively generated by passive Na^+^ uptake from the environment leading to a trans-epithelial potential (TEP) difference. Differences in TEP between various regions form intra-embryonic voltage gradients. The magnitude of the arising endogenous EF is in the order of 1–5 V/cm and thereby above the minimum level needed to affect morphology and cell migration of embryonic cells *in vitro* (see chapter “Migration”) (Hotary and Robinson, 1990; Metcalf et al., 1994; Levin, 2007).

Not only small ions like protons, sodium or potassium can be involved in EF patterning, but also larger biomolecules like tissue factors, growth hormones, transmitters and signaling molecules like serotonin and others—nearly all possess electrical charges besides their chemical and receptor mediated action (Vandenberg and Levin, 2012). All these factors carry information for a single cell but also for the neighboring cells. Thus, an extreme complex picture emerges, “drawn” by cell- and molecular biological methods, which gathered a tremendous amount of new data in the recent decade.

Taken together, the membrane potential—generated EF is possibly the first and most subtle hitherto detectable general biological information system, at least with recent technologies. It is suggested that along with the emerging availability of oxygen about 1.5 billion years ago, primitive organisms could have developed energy-fueled transport systems for nutrition and osmoregulation (Franciolini and Petris, 1989). Later the hitherto intracellular messenger Ca^2+^ could have served as a signaling messenger from outside the cell to inside. This probably happened in a common ancestor of Archaebacteria and Eubacteria as mechano-sensory calcium channels (Franciolini and Petris, 1989). These channels shifted the membrane potential away from being neutral, thereby building up a charged membrane potential. Thus, it is conceivable that this established a first general information system before hormones or nervous structures etc. evolved. We must discern these general electric information that appear early in evolution from the special electric information which appeared later as a guiding cue for cells in a multi-cue environment (e.g., in neural crest migration—see “Outlook”). In vertebrates connexins (gap junction components, see below) are first detected at the 8—cell stage (Houghton, 2005), and gap junctions can contribute to conveying electrical information (see below).

Why was this obvious concept of EF fields for coding information ignored for such a long time in biology, and why is it still not integrated into modern molecular and cell biology? First, this was caused by a misuse and charlatanism regarding “electro-stimulatory” apparatuses from the end of the 19th century on (see history of “bioelectricity” from Galvani on in the reviews of McCaig et al., 2005; Levin, 2007; Funk et al., 2009; Funk, 2013). Second, this is due to the lack of appropriate methods for measuring the EF, because in the beginning only single electrode measurements were possible (DuBois-Reymond, Burr, Becker and Nordenström—see the reviews mentioned above). The next big step was the development of small vibrating, self-calibrating electrodes (Jaffe and Nuccitelli, 1974; Hotary and Robinson, 1992; Hotary et al., 1992; Reid et al., 2007), which could measure EF and related currents in a sub—millimeter dimension and could really tackle the EF to biologically processes. Third and finally, it is the field concept as such which makes it hard to handle in the molecule- and cell-centered “classical” cell biology (Driesch, 1929). “Field” reminds of “morphogenetic fields,” or “subtle fields,” e.g., found in esoteric literature for modern molecular biologists -phenomena which are not to grasp and are difficult to “nail down.” This is, however, now possible with endogenous EF: they can be observed with modern *in vivo* dyes directly in cell- and even in sub-cellular dimensions (see also the reviews mentioned above). And since the last decade it is possible to link these EFs directly to “hard—core” molecular biology of the cell. The following examples will show this:

**Functional roles of endogenous electric fields (EF)**: Domains of distinct membrane potentials can be observed in single cells (Adams and Levin, 2013) from the fertilized egg on and also from the electric (!) and then by calcium wave mediated polyspermia block on Grey et al. (1982), triggering the neighboring cell to build up an electric field. Later on these fields spread over the early **embryo**. Such EFs are in general the first information cues that determine domains like anterior/posterior or left/right in the very early embryo, e.g., in the flatworm (*planaria*) (see Oviedo et al., 2010; Beane et al., 2013). Even within the circumference of a plasma membrane in unicellular organisms (*protozoa*), regular and sharply confined patterns could be found that define, e.g., the position of cilia and other features (Adams and Levin, 2013).

These EF, which *per se* are also able to de- or hyperpolarize the membrane potential, subsequently induce the expression of signaling factors, so that finally morphological patterns arise that can be observed as distinctive folding, proliferation and migration of different cell groups (see below). A disruption of this information cascade during development, e.g., by blocking of cell membrane bound proton transporters, will ultimately lead to malformation, dislocation of organs and other severe defects (Zhao et al., 2006; Levin, 2011, 2012). EF and ion flows are tightly involved in developmental differentiation control. Areas of similar EF confine related cell groups of iso—electric and iso—pH value. It may also be possible that the same frequency of UL EMF patterns (see “Outlook”) represents this common denominator. It is well documented that vertebrate embryos possess steady voltage gradients, particularly in areas where major developmental events occur in relation to cell movement and cell division (Vandenberg et al., 2012). Importantly, these electric field patterns precede major morphological events in development, e.g., electric currents precede and predict the point of emergence of the limb bud by several days in the amphibian embryo pre-limb bud region (Altizer et al., 2001; McCaig et al., 2005; Levin, 2014). Transepithelial potential differences in the early embryo are the source of current loops that were detected with non-invasive vibrating electrodes. Outward currents are found at the lateral edges of the neural ridges and at the blastopore, whereas inward currents are found at the center of the neural groove and at the lateral skin (see Shi and Borgens, 1995). Here, an endogenous “self-electrophoresis principle” may operate around the cell membranes where an external back current ought to close the current loop, as could be shown by membrane potential modulation (see De Loof, 1986, 1992; De Loof et al., 2013). The self-electrophoresis principle means that under these conditions the cells shift charged molecules, thereby driving an electrical current.

A good example of major patterning events is left—right patterning, because it directs the position of organs like heart, liver and organs of the digestive tract asymmetrically to the left—right axis. This example shows, how the subtle field information of the EF is transferred to more fixed biochemical signaling pathways and transferred down to the genome level, finally ending in morphological patterning (Adams et al., 2006; Carneiro et al., 2011). Distribution of cell membrane components begins already in the fertilized egg and ends in an asymmetric distribution of ion channels and pumps. This can lead to asymmetric ion gradients (Levin et al., 2002; Adams et al., 2006), which subsequently drive the establishment of physiological gradients of molecules (e.g., serotonin) (Fukumoto et al., 2005a,b). After the first embryonic cell divisions the cells on the right side are more negatively charged due to the polarized distribution of ion gradients. A network of open gap junctions then distributes left–right signaling molecules to the right and most ventral blastomere (Adams and Levin, 2013). These signaling molecules ultimately control the expression of asymmetric genes by a histone deacetylase (HDAC)-dependent intracellular receptor (Carneiro et al., 2011). Thus, HDAC activity is a left–right determinant in controlling the epigenetic state of defined genes at the early developmental stages. The HDAC binding partner Mad3 may then be the new serotonin-dependent regulator of asymmetry, linking early physiological asymmetries to stable changes in gene expression during organogenesis (Carneiro et al., 2011). This process sets the foundations for an asymmetric Anlage of cilia and thereby also the direction of the cilium beat. Furthermore, the other body axes (anterior—posterior and ventral—dorsal) are preformed in a similar cascade of events and further patterning of substructures. Electrophysiological parameters were also found for craniofacial patterning in *xenopus* embryos: Vandenberg et al. (2012) observed a complex pattern of voltage gradients, driven by the regionally different activities of the V—ATPase (Vacuolar-type H^+^ -ATPase) proton pump at the primitive oral opening and the neural tube. Interestingly, a perturbation of voltage domains and pH gradients results in changes of gene expression which drive this orofacial patterning, resulting in malformations.

Regarding early embryonic development and subsequent cell migratory pathways, multiple possibilities of conveying, storing and transducing information into classical cell and molecular pathways exist: First, they exist by the magnitude of the membrane potential of the cell itself. Second, different domains with distinct resting membrane potentials can exist, as an example, they can be driven by different transporters, exchangers etc. within dendrites and axons of nerve cells. These different domains can even be observed in unicellular organisms, e.g., where the position of cilia is indicated by these membrane potential domains (see Adams and Levin, 2013). Third, different frequencies of membrane potential fluctuations exist like (outlined above). Fourth, different types of ions and charged biomolecules like histamine convey different streams of information recognized by different transporters, exchangers and receptors on the cell membrane. Functional implications of the role of endogenous electric fields in tissues, organs and the whole body do not only influence embryonic development, but also tissue and organ formation (Shi and Borgens, 1995), or regeneration and wound healing (see McCaig et al., 2005), cell migration (Zhao et al., 2006; Ozkucur et al., 2009, 2011), and cell differentiation and proliferation (see Vandenberg and Levin, 2012) (for review see also literature in Funk et al., 2009; Adams and Levin, 2013).

During **wound healing** enhanced DC- EF are present: epithelial layers exhibit a TEP and immediately upon being wounded this TEP is enhanced at the cathode (originating endogenously by a breach in the epithelial layer) at the wound center. Ion transporters play a significant role in producing the wound induced electric potentials. Here, Sun et al. (2011) could show in airway epithelial wounds that inhibiting the cystic fibrosis transmembrane conductance regulator significantly reduced wound currents. The EF is then probably the earliest signal that an epithelial cell receives to initiate directional migration into the dermal wound bed (Nuccitelli, 2003; Ojingwa and Isseroff, 2003). Even transient breaches in an epithelial layer, which do also occur during physiological epithelial turnover, induce short-lived, local electrical signals that influence cell regulation (see McCaig et al., 2005). However, wound-induced electrical signals can last for many hours and days (see McCaig et al., 2005). These signals regulate different cell behavior within 500 μm to 1 mm apart from the wound edge. The signal then fades after complete covering of the wound by the epithelium. Interestingly in corneal epithelial wound healing, the electric field lines do even control the orientation of the mitotic spindles in proliferating epithelial cells and also the orientation of re-growing nerve sprouts (Song et al., 2002, 2004). Similarly, in cultured hippocampal neural and glial cells the cleavage planes were oriented perpendicular to the EF field lines (Yao et al., 2009). Regarding the time course of corneal epithelial wound healing *in vivo*, Kucerova et al. (2011) showed that the electric fields first trigger only the initial signals after wounding, like planar polarization of the cells, while other factors like growth factors etc. take over later on. Finally, EF induce gene activation takes over, e.g., Zhao et al. (2006) could show that during wound healing phosphatase and tensin (PTEN) homolog enzymes are directly involved.

This is a good example to show that electrical short-circuiting must be the fastest signal and the diffusion of signaling molecules and hormonal actions are slower and occur later. This points out that EF bridge the information gap between short-range molecular actions of local hormones, e.g., exocrine glands or growth factors, and between long-range actions of hormones, e.g., endocrine hormone distribution via the blood flow and long-range actions of the nervous system.

Here, Nogi and Levin (2005) suggested that gap junctional communication may be required for long-range anterior/posterior patterning in *planaria*. Oviedo et al. (2010) could indeed show in this species that gap junctions are required for signaling long—range information on anterior/posterior body axis to the blastema during regeneration. In this case, information spreads over many hundreds, if not thousands, of cells (see below for further explanation on how this might work). Still, more studies are needed to show how this occurs in larger animals including also mammals, where it is known for local areas, e.g., during craniofacial patterning in *xenopus* embryos (Vandenberg et al., 2012). Finally, studies that show a possible cooperation between gap junctional signaling and signaling of the nervous system remain to be conducted. Regarding the link to classical cell signaling pathways, it was shown that ion gradients which are produced by membrane potentials can drive charged molecules like serotonin (see Levin, 2007; see also text below). In addition, serotonin is also able to traverse gap junctional paths under an electrophoretic force (Levin, 2009a,b). Besides serotonin signaling, bioelectric cues can couple into classical cell biological pathways via calcium (see above), phosphatases (PTEN, see above), or the chromatin modification machinery (Carneiro et al., 2011).

With respect to **regeneration**, experimental models like limb regeneration in salamanders and newts (Borgens et al., 1977; Borgens, 1984; Altizer et al., 2002) encompass so many facets that it is currently quite difficult to unravel all molecular aspects of ion transporter locations. Recently, our group (Ozkucur et al., 2010) demonstrated that ion contents in the axolotl tail blastema change dynamically during regeneration and, in most cases, are still fluctuating 48 h after amputation. More detailed, after 6 h the membrane potential was depolarized by five-fold in the bud region blastema compared with other regions and the uncut tail. Especially the epidermal and mesenchymal cells were involved in these early events with both having elevated membrane potentials compared with, e.g., melanophores. After 24 h the cells in the bud region had a relatively high pH and extruded protons (see also Adams et al., 2006). Adams et al. (2007) found in the amputated *xenopus* tail model a V-ATPase-dependent proton extrusion already 6 h after amputation. The proton extrusion induces an EF with the negative pole being outwardly directed, which is large enough to induce the directed growth of nerves of the spinal cord. These processes were followed by an increased Na^+^ flux in the regenerating bud and after 24 h activation of downstream pathways (BMP, Notch, Msx, Wnt, and Fgfs) (Tseng et al., 2010). Regeneration is then completed after 7 days. Artificial modulation of wound physiology by addressing the right ion transporters may therefore be a promising approach for augmenting and inducing regeneration in otherwise non-regenerative tissues. Interestingly, serotonin often transmits bioelectric signals into classical signaling pathways, e.g., in left/right patterning (see above). Especially with regard to stem cells, further EF studies in the context of regeneration are needed.

## Spreading of information in EF and ion gradients

How can electric fields spread over distances up to millimeters in an embryonic or adult organism? One possible way is within the extracellular space similar to pH gradients (Ozkucur et al., 2010; Schreml et al., 2014). Another important way is spreading via gap junctions, which are also termed “electrical synapses” in neurosciences (Pereda, 2014). Special membrane proteins build up a certain channel or junction between neighboring cells to form a gap junction. These membrane proteins are called connexins in vertebrates and innexins in invertebrates. The innexins have recently be renamed pannexins, because they were recently also found in vertebrates. Gap junctions can convey not only molecular, but also electric information directly from one cell membrane to the next one (see below). Thus, groups of cells can exchange ions (e.g., Ca^++^, K^+^), second messengers like cAMP, cGMP or IP3 or metabolites very rapidly, thereby building up gradients in their membrane potentials and thus EF very quickly (Pereda, 2014). Gap junctions connect nearly all cell types, and interestingly connect also cells which are not residing in close proximity, by extended processes that often also possess gap junctions (Wang et al., 2012). Even in cells which project tunneling nanotubes (TNTs)—very thin and long processes with diameters between 1 μm and 100 nm—such gap junctions are integrated, e.g., to transport Ca^++^ ions (see Wang et al., 2010; Wittig et al., 2012). By this cells can stay in contact even in tissues with a high content of extracellular material, as it was shown *in vivo* for corneal tissue (Chinnery et al., 2008). Before discovery of TNTs similar contacts were found in cells of tendons (McNeilly et al., 1996). Interestingly, such TNTs can also be observed after cell division in cell or tissue cultures. Dividing cells which move apart can stay in contact over a distance of up to 1 mm via TNTs. Thus, this “TNT—leash” is supposed to be very important also during migratory processes, e.g., during migration of the neural crest cells, although this remains to be proven. Moreover, processes of migrating cells can also transmit, e.g., Wnt (Wingless and Int1 –coded by corresponding genes) signaling over long distances (Levy-Strumpf and Culotti, 2014), but the question remains from which guidance cue these processes get information where to go? This again foregrounds the easiest and fastest way to build up a gradient: the endogenous EF. In EF, membrane potentials can be coupled to initiate a first coherent information wave, e.g., via gap junctions. This gradient can also be sensed by cells which migrate onto an electrically coupled cell layer similar to axonal path finding via the growth cones of neural cells. Excellent studies and review articles of Borgens and the McCaig group describe this phenomenon of EF directed axonal guidance of neurons (see McCaig et al., 2005, 2009).

## Migration

Migration of cells is constitutive for tissue and organ formation and is also important for regeneration and wound healing. Interestingly, bioelectric factors override most chemical gradients. *In vitro* experiments revealed that many cell types prefer to migrate to the cathode when the externally applied field strengths is around 0.1–10 V/cm (electrotaxis), e.g., neural crest cells, fibroblasts, keratinocytes, chondrocytes, rat prostate cancer cells, and many epithelial cell types (Robinson, 1985; Nishimura et al., 1996; McCaig and Zhao, 1997; Zhao et al., 1997; Djamgoz et al., 2001; Pullar et al., 2006). In contrast, only few cell types move to the anode, among them corneal endothelial cells, bovine lens epithelium, human granulocytes, and human vascular endothelial cells (see Funk et al., 2009). Both speed and direction of the movement are voltage dependent. Electrotaxis as the movement of cells along an electric field gradient is further modulated by species and cell subtype differences. For example, SAOS (an osteosarcoma cell line) migrate in the opposite direction than rat calvaria osteoblasts in primary cell cultures (Ozkucur et al., 2011).

This opens up the question what are the mechanisms behind the directed movement of a cell in an electric field gradient? First there should be charges on the cell membrane surface which are either mobile or which are able to translate into a reorientation of the cell (electrophoretic mechanism, Lammert et al., 1996). Furthermore, if the guidance cue, e.g., a gap in an epithelial layer, is distant, a persistent movement has to be achieved. Furthermore, all these interactions need to be reapplied periodically until the cell has arrived at the correct site. All molecular components which are needed for persistent directional cell migration are currently analyzed in detail. The simplest signal is the unidirectional signal of a linear breach in an epithelial layer during wound healing (see above). Here, the signal of the wounding gradient must be sensed first at the cell membrane, either by charged molecules which then indirectly leads to grouping of receptors for these molecules at the site of influx of the plasma membrane circumference, or more probably by a reorientation of charged molecules at the cell membrane (electrophoretic redistribution of membrane components, Allen et al., 2013) which is directly elicited by the field gradient itself (see Kindzelskii and Petty, 2005). In *Dictyostelium* cells, Gao et al. (2011) could show that at the cell membrane the initial directional sensing mechanisms for electrotaxis differ from those of chemotaxis. Na^+^/H^+^ exchanger (NHE) isoforms are located in the cell membrane and in intracellular organelles of mammals (Donowitz and Li, 2007). NHE1 regulates intracellular pH and volume, while NHE3 is involved in Na^+^ reabsorption and proton secretion (Wheatly and Gao, 2004).

Indeed, differences in pH between the front and the rear end have been observed in migrating cells, and inhibition of NHE1 decreased or even dissolved the pH gradient. Hence pH gradients produced by NHE1 are present along the axis of movement (Martin et al., 2011). Besides electrotaxis, NHE3 might also be relevant to all directional migration processes under a constant cue with the cell membrane potential probably acting as a regulatory element. In our previous study, we reported on patchy accumulations of physiologically active pNHE3. In addition, we observed H^+^ bubbles outside the cell membrane particularly at the leading edge of migrating cells, and also a ß-actin accumulation inside the membrane (Ozkucur et al., 2011). In the presence of a persistent external EF, constant directionality was impaired when NHE3 was silenced in osteoblasts. The cells lost their characteristic polarity and orientation (Murphy and Courtneidge, 2011). We found that the directional information of NHE3 is transferred via a mechanism that involves PIP2 as a potential mediator for maintaining a pathway for electrotaxis (Ozkucur et al., 2011). Interestingly, Sun et al. (2013) proposed a compass model of electro- (galvano-) taxis with their observation that PIK3—and myosin-dependent pathways perform differently in fish keratocytes and corresponding cell fragments—PI3Ks being responsible for the production of phosphatidylinositol 3-phosphate (PIP3).

**Figure 1:**
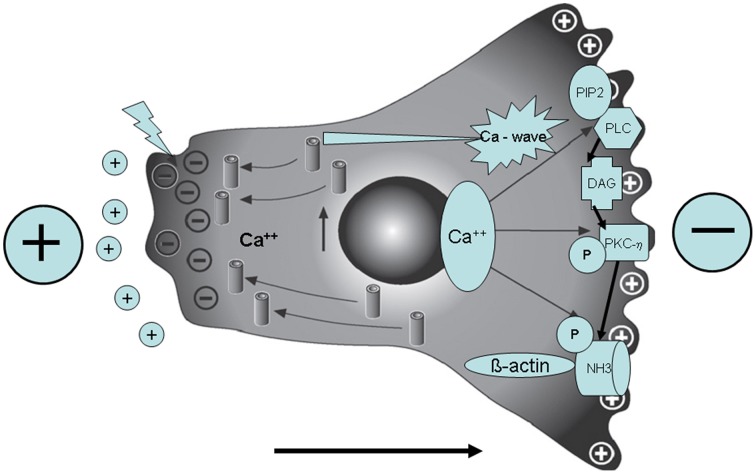
**In the presence of a directional cue (external electric field—polarity see big + and −) there is a depolarization of the rear end of cell movement (big arrow; bottom), then a Ca^++^ influx and a Ca^++^ wave to the front end**. In addition NHE3 is activated via PKC. Note the pNHE3/betaactin complex formation (here: primary osteoblast in an artificial external electric field in the physiological range applied by chamber electrodes).

What is more, we found in mammalian cells that pNHE3 forms complexes with both PKCη and γ-tubulin at filopodia, suggesting that these molecules may regulate the microtubule-organizing center. Our data further suggest that PKCη-dependent phosphorylation of NHE3 and the formation of pNHE3/PKCη/ γ-tubulin complexes at the leading edge of the cell are required for directional cell migration in an EF (see Figure 1) (Ozkucur et al., 2014; Perike et al., 2014). Thus, the molecular details of the translational force of EF that induce transporters, cytoskeletal elements and migration were unraveled.

## Outlook

Regarding migration there is mounting evidence that EF are driving factors in cell migration that often override other cues in a multi–cue environment. Thus, from the early embryonic development on, many steps in further cell differentiation and cell migration should be revisited in the light of these new findings. Current models in textbooks explain the regulation of body axis specification by a Cartesian coordinate system of the distribution of signaling proteins like Wnt and bone morphogenetic protein (BMP) (Niehrs, 2010). In this context, the cooperation of charged signaling molecules driven within an electric field and the role of the EF itself, e.g., for neural crest migration, should be analyzed in further studies, because this opens up a pool of many exciting topics for future research. The timing and order of different levels of morphological fixation (1. electrical, 2. biochemical, 3. definitively morphological) has to be considered, because these steps occur subsequently and in interlacing patterns, and must be timed exactly. Another open question is whether these bioelectric fields are more static (at least they following embryonic steps within the range of minutes and hours), or if these fields are fluctuating faster in electromagnetic waves, at least at very low frequencies of 0.05—several Hz. There are some hints that this is very probable, but this needs to be proven in future studies. Young and Rozengurt (2010) could show fluctuations of about 0.05–0.1 Hz during receptor signaling by Ca^++^—imaging in pancreatic cancer (PANC-1) cells. Raimondo et al. (2013) could demonstrate activity-dependent fluctuations in pH of about 0.2 Hz in hippocampal cells.

What is more, within the interior of a cell, organelles like mitochondria synchronize with the membrane potential to neuronal activity and to Ca^++^—waves (Kovács et al., 2005). Likewise, the enzyme machinery within the cell does not work in a linear manner, as proposed by Rosenspire et al. (2005), who discuss an electrically sensitive, membrane-embedded receptor complex such as VSP, which transduces the signal to 1–25 Hz Ca^++^ pulses. The frequency of the calcium pulses must be compared with the fundamental 0.05 Hz metabolic oscillations. Rosenspire et al. (2005) argue that “the intermediate metabolism of the cell functions as a biochemical bandwidth filter centered at 0.05 Hz. In this way, the 0.05 Hz electrical pulse-frequency domain of interest is seen to arise quite naturally.” However, the “electric” interior of the cell is difficult to measure and is therefore yet to be mapped. To date, only few and initial attempts were made to analyze intracellular fields of organelles like mitochondria, e.g., using “nano—pepple” sensors (Tyner et al., 2007; Lee and Kopelman, 2012).

To sum up, this implies that multicellular systems cannot forward only static electric “information” by gap junctions or other cell-derived structures, but they are also able to “swing in” by coherent rhythms to initiate cell functions like self—protection, proliferation, or apoptosis. Coming back to embryogenesis, Adams and Levin (2013) have noted that spatially periodic, temporally stable patterns arise from the instability of a homogenous steady state (first mentioned in a classic paper of Turing, 1952), and that these represent a mechanism for embryonic development.

In the case of long-range intercellular information (e.g., by gap junctions), this should mean that small local sources of membrane potential changes (symmetry breaking points) can lead to recursive feedback information from the whole cell array and lead to field like patterning of, e.g., cell differentiation. Like it is found in “metabolic patterns” (“physiological gradients,” Child, cited by Schiffmann, 2008) were patterns arise by metabolic activators and inhibitors (similar to Turing-Gierer-Meinhardt model) which disappear when the flow of electrons is inhibited (Schiffmann, 2006, 2008).

This subtle patterning information of gap junction transferred electrical information (1st level) must then be transduced into the genetic machinery - inducing specific gene expression (see Adams and Levin, 2013). This can happen like described above, e.g., by controlling the distribution of signaling molecules like histamine (2nd level), which control the expression of asymmetric genes (3rd level) by a HDAC-dependent intracellular receptor.

However, to evolve a complete organism from a fertilized egg, these cascades of events have to be recursive to the genome and back in the different layers of organization, being another further challenge for bio–informatics and (fractal and other) bio–mathematics—many fascinating topics for future research. The described electrically guided cell migration is only one process, however, a very important one.

### Conflict of interest statement

The author declares that the research was conducted in the absence of any commercial or financial relationships that could be construed as a potential conflict of interest.
